# Phylogenetic Lineages and Diseases Associated with *Moraxella catarrhalis* Isolates Recovered from Bulgarian Patients

**DOI:** 10.3390/ijms25189769

**Published:** 2024-09-10

**Authors:** Alexandra S. Alexandrova, Vasil S. Boyanov, Kalina Y. Mihova, Raina T. Gergova

**Affiliations:** 1 Department of Medical Microbiology, Medical Faculty, Medical University of Sofia, 1431 Sofia, Bulgaria; v.boyanov@medfac.mu-sofia.bg (V.S.B.);; 2 Department of Medical Chemistry and Biochemistry, Molecular Medicine Center, Medical Faculty, Medical University of Sofia, 1431 Sofia, Bulgaria

**Keywords:** *Moraxella catarrhalis*, respiratory tract infections, molecular epidemiology

## Abstract

*Moraxella catarrhalis* has been recognized as an important cause of upper respiratory tract and middle ear infections in children, as well as chronic obstructive pulmonary disease and chronic bronchitis in adults. We aim to study the clonal structure, antimicrobial resistance, and serotypes of *M. catarrhalis* strains recovered from patients of different ages. Nasopharyngeal swabs, middle ear fluid, and sputum samples were collected. In vitro susceptibility testing was performed according to EUCAST criteria. The monoclonal Ab hybridoma technique was used for serotyping. All strains were subjected to MLST. The studied population demonstrated susceptibility to all tested antimicrobials *M. catarrhalis* strains, with the majority being serotype A (90.4%), followed by B (6.8%), and C (2.7%). We observed a predominant clonal complex CC224 (21.9%) along with other clusters including CC141 (8.2%), CC184 (8.2%), CC449 (6.8%), CC390 (5.5%), and CC67 (2.7%). Two primary founders, namely, ST224 and ST141, were identified. The analyzed genetic lineages displayed diversity but revealed the predominance of two main clusters, CC224 and CC141, encompassing multidrug-resistant sequence types distributed in other regions. These data underscore the need for ongoing epidemiological monitoring of successfully circulating clones and the implementation of adequate antibiotic policies to limit or delay the spread of multidrug-resistant strains in our region.

## 1. Background

*Moraxella catarrhalis* is a species in the genus *Moraxella*, Phylum *Pseudomonadota*, Class *Gamma proteobacteria*. It is an aerobic, nonmotile, nonspore-forming, Gram-negative diplococcus that is positive for oxidase, catalase, and indoxyl acetate esterase [1]. *M. catarrhalis* has been recognized as an important cause of otorhinolaryngological (ORL) infections in childhood, as well as chronic obstructive pulmonary disease (COPD) and chronic bronchitis in adults. The clinical interest in *M. catarrhalis* is relatively recent, and many laboratories in the past did not report *M. catarrhalis* as a pathogen, especially in cases when other well-known pathogens are present in the same specimens [1]. The carriage rate of *M. catarrhalis* in children is high, reaching up to 60% in some regions [2,3,4]. In childhood, nasopharyngeal colonization often precedes the development of *M. catarrhalis*-mediated disease, mostly after viral infection [5,6]. Authors have observed seasonal variations in *M. catarrhalis* infections, [7,8] with the highest incidence occurring in winter and spring [9,10,11]. Highly virulent *M. catarrhalis* strains can be the single cause of serious infections, such as sinusitis, otitis media, tracheitis, bronchitis, and pneumonia, and, less commonly, ocular infections in children [12,13,14,15,16,17,18,19,20,21]. Occasionally, the bacterium causes systemic disease, e.g., meningitis and sepsis [22,23,24,25]. Bacteremia due to *M. catarrhalis* should be considered, especially in febrile children with compromised immune function after a heavy ORL infection [26,27].

Different components in the outer membrane, such as proteins UspA2, OmpB1, OmpB2, OmpCD, OMPE, and lipooligosaccharides (LOSs), which mediate adhesion and complement resistance, are considered important virulence factors in the pathogenesis of *M. catarrhalis*; these factors enable the bacterium to surmount a major mechanism of human immune defense and have immunogenic properties [21,28,29]. The initial stages of bacterial colonization and infection involve interactions of the CD protein of *M. catarrhalis,* which has been shown to specifically attach to the mucin molecules from the nasopharynx and middle ear but not to mucin from saliva and tracheobronchial mucin [30,31]. OMPE is considered a major adhesion factor in human respiratory tract mucosal epithelium colonization by *M. catarrhalis* strains and is also involved in the transport of fatty acids [21]. There is no definite consensus on the presence or absence of fimbriae and its influence on the capacity of *M. catarrhalis* to adhere [32]. Lipid A from *M. catarrhalis* LOSs, also found in the cell walls of other species, has biological activity as an endotoxin. It can cause fever; block the complement system; stimulate the immune system by binding to Toll-like receptors; and lead to disseminated intravascular coagulopathy (DIC), shock, adrenal hemorrhage, and death [33,34].

According to the LOSs in the outer membrane, over 95% of *M. catarrhalis* strains can be classified into three serotypes: A, B, and C. The most common serotype is A, accounting for about 60% of the strains, while serotypes B and C are less prevalent, representing approximately 30% and 5% of the strains, respectively. Around 5% of *M. catarrhalis* strains cannot be serotyped [35].

*M. catarrhalis* is generally considered to have high sensitivity to antibiotics. The common antimicrobial resistance in *M. catarrhalis* is to penicillin due to the production of BRO β-lactamases. In *M. catarrhalis*, two types of β-lactamases can be found that are phenotypically identical: BRO-1 and BRO-2 [3,36]. Both are membrane-associated, and they differ by only a single amino acid. The enzymes are encoded by chromosomal genes, and these genes can be relatively easily transferred from cell to cell by conjugation [37,38].

Genotyping analysis and the establishment of genetic lineage relationships of *M. catarrhalis* are insufficient, particularly in Europe. In epidemiological studies, multilocus sequence typing (MLST) and whole genome sequencing (WGS) are employed to uncover the clonal structure and relatedness with internationally recognized clones [39,40].

We aim to study the clonal structure, antimicrobial resistance, and serotyping of *M. catarrhalis* strains isolated from patients of different ages with upper and lower respiratory tract infections collected during the 2024 winter–spring season in Sofia, Bulgaria.

## 2. Results

### 2.1. Studied Population

The 73 examined *M. catarrhalis* strains were collected from the Department of Medical Microbiology at the Medical University of Sofia during the 2023–2024 winter–spring season from patients with ages ranging from 1 to 74 years old (Table 1). They were categorized into two age groups: children from 1 to 11 years and adults from 50 to 74 years old. The most common age group was 1–5 years old, accounting for 41 (56.2%) cases. The young patients were diagnosed with respiratory tract infections, including rhinopharyngitis, rhinosinusitis, and adenoiditis (n = 34, 82.9%), as well as otitis media (n = 7, 17.1%). Among adult patients (50–70 years old), cases of chronic bronchitis and COPD totaled 20 (27.4%). Regarding gender, there were 46 (63%) male patients and 27 (37%) female patients.

### 2.2. Serotyping

Serotyping using MAbs revealed that 90.4% (n = 66) of *M. catarrhalis* strains were serotype A. Serotypes B and C were represented by n = 5 (6.8%) and n = 2 (2.7%) strains, respectively.

### 2.3. Antimicrobial Susceptibility

**Figure 1:**
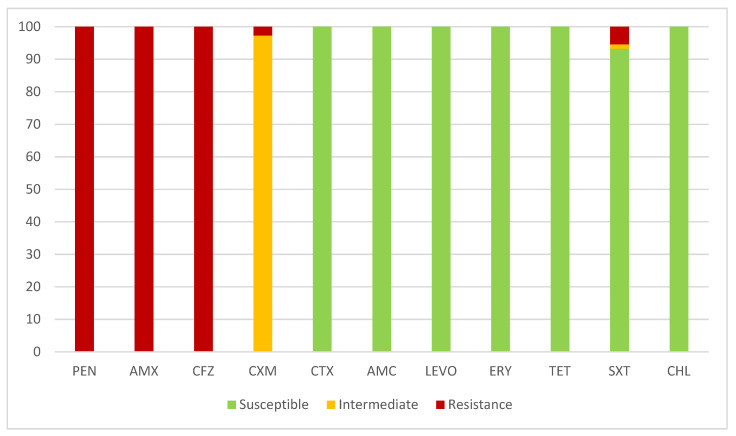
Antimicrobial susceptibility of 73 *M. catarrhalis* strains isolated from Bulgarian patients in the winter–spring season of 2023–2024. PEN—Benzylpenicillin, AMX—Amoxicillin, CFZ—Cefazolin, CXM—Cefuroxime, CTX—Cefotaxime, AMC—Amoxicillin/Clavulanic acid, LEVO—Levofloxacin, ERY—Erythromycin, TET—tetracycline, Sxt—Trimethoprim-sulfamethoxazole, CHL—Chloramphenicol. The interpretation was performed according to the European Committee on Antimicrobial Susceptibility Testing (EUCAST 2024) breakpoints.

All the strains demonstrated resistance to penicillin, aminopenicillins, and the first generation of cephalosporins due to the presence of BRO β-lactamases detected using the Cefinase disc method. However, the strains exhibited very high susceptibility to almost all other tested antibiotics (Figure 1). The strains produced BRO-1 β-lactamase, and only one strain produced BRO-2 β-lactamase.

All *M. catarrhalis* strains exhibited intermediate susceptibility to oral cefuroxime, and two strains were resistant. A small number of strains (n = 4, 5.5%) were resistant to trimethoprim-sulfamethoxazole or exhibited intermediate susceptibility (n = 1, 1.4%). None of the strains (0.0%) were resistant to amoxicillin/clavulanic acid, cefotaxime, erythromycin, tetracycline, chloramphenicol, or levofloxacin (Figure 1).

### 2.4. Genotyping

The MLST analysis of the patterns of all 73 *M. catarrhalis* isolates revealed 36 STs, including one novel ST (STN).

In total, 39 strains (53.4%) exhibited similarity with different STs and formed a clonal complex in which every ST shares at least five of eight identical alleles with at least one other ST. They form the main clonal complexes in the studied population. The distribution among the STs showed that ST224 (n = 9, 12.3%) was predominant. In total, 36.9% of STs were represented by three strains each, including ST54, ST62, ST141, ST184, ST195, ST216, ST390, ST435, and ST449. In addition, 27.4% of the STs were found in two strains each, including ST3, ST183, ST215, ST227, ST436, ST498, ST540, ST952, ST1022, and STN.

**Figure 2:**
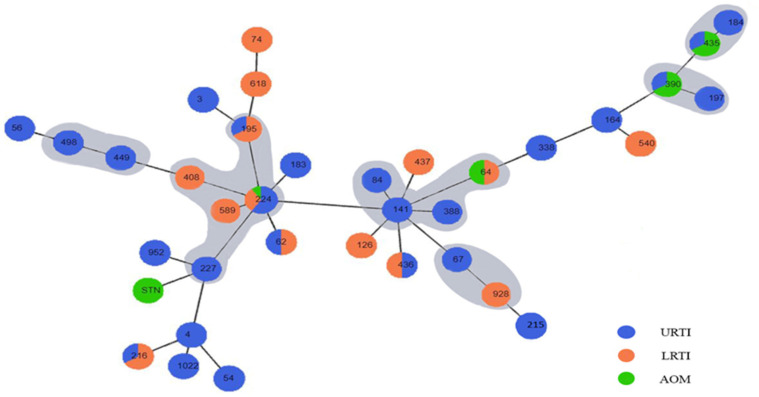
Population snapshot of 73 *M. catarrhalis* strains recovered from children and adults with respiratory infections in Bulgaria. The sequence types (STs) are identified based on the allelic profiles on the MLST website and depicted inside the circles. The clonal complexes (CCs) were named after the predominant STs. If the STs are in an equal number, the CCs were named after the earlier STs found in the population. The clonal complexes are surrounded with a grey halo and comprise STs with relatedness at different levels: SLVs (single locus variants), DLVs (double locus variants), and TLVs (triple locus variants). The lines between the STs correspond to the relatedness. Specifically, the shortest lines correspond to SLVs. The longer the line is, the more genetically distant the representatives are. The diameter of the circles corresponds to the number of the STs. The colors in the circles correspond to the diagnosis of the patients: URTI—upper respiratory tract infections: rhinopharyngitis and rhinosinusitis, LRTI—lower respiratory tract infections: COPD (chronic obstructive pulmonary disease) and bronchitis. AOM—acute otitis media. The diagram was constructed using Phyloviz 2.0.

We identified a predominant clonal complex CC224 (n = 16, 21.9%) and other small clusters, including CC141 (n = 6, 8.2%), CC184 (n = 6, 8.2%), CC449 (n = 5, 6.8%), CC390 (n = 4, 5.5%), and CC67 (n = 2, 2.7%). Two primary founders, namely ST224 and ST141, were disclosed. Within CC224, the ancestral ST224 revealed high similarity to ST408 and ST589 with a difference of one locus, and ST227 and ST195 were double and triple locus variants, respectively, displaying six and five identical alleles (Figure 2). 

The other primary founder ST141 was clustered with ST64, ST84, and ST388, which shared seven or five identical alleles, indicating SLVs or TLVs. The ancestral types ST224 and ST141 were discovered both in children and adult patients with upper and lower respiratory tract infections. 

CC184 represented six strains isolated from patients of different ages and diagnoses and clustered with ST184 and the SLVs ST435. CC449 is comprised of STs and its TLV ST498. ST390 and ST197 formed CC390. ST67 and ST928 displayed five identical house-keeping genes and comprised CC67. 

We also observed that 32.9% of the strains formed small clusters of two or three isolates with the same sequence type (ST), but they did not share similarities with any other STs in the studied population (Figure 2). These individual STs were not common among the analyzed strains and did not show any relatedness to other STs. However, we anticipate successful spreading in the coming years of this type of clonal complex, which is exclusively composed of a single sequence type. These small groups were represented by ST216 (n = 3, 4.1%), ST54 (n = 3, 4.1%), ST3 (n = 2, 2.7%), ST62 (n = 2, 2.7%), ST183 (n = 2, 2.7%), ST215 (n = 2, 2.7%), ST436 (n = 2, 2.7%), ST540 (n = 2, 2.7%), ST952 (n = 2, 2.7%), ST1022 (n = 2, 2.7%), and STN (n = 2, 2.7%).

The STs that showed no relatedness to any other STs and were considered singletons accounted for 13.7%.

All clustered strains and singletons along with their main phenotypic and genotypic characteristics are presented in Table 2.

## 3. Discussion

It has become evident over the past few decades that *M. catarrhalis* has significant pathogenic potential. The bacterium is mainly involved in respiratory tract infections and rarely in systemic infections in immunocompromised patients. Many authors considered *M. catarrhalis* one of the most common bacterial organisms that causes otitis media, following the nontypeable *Haemophilus influenzae* (NTHi), *Streptococcus pyogenes*, and *Streptococcus pneumoniae* [41,42]. In our study, the number of cases of otitis media was lower compared to other upper respiratory tract infections, as we did not collect specimens only from a specific group of patients diagnosed with AOM, but in general, from patients with respiratory infections caused by *M. catarrhalis*.

The majority of the strains that were examined were found in young children. Many studies have reported that *M. catarrhalis* colonizes infants and children at a higher rate, especially in those under 5 years of age, with only 1–5% carriage in healthy adults. However, isolates from older patients were more likely to be pathogenically significant [18,43]. In our study population, all strains that caused chronic bronchitis and COPD were found in adults, while all cases of acute otitis media (AOM) were seen in children, mostly those under 5 years old.

Our results showed that all *M. catarrhalis* strains were resistant to penicillin, aminopenicillins, and first-generation cephalosporins and exhibited lower or no susceptibility to cefuroxime oral. The results demonstrated a progression of elevation of resistance to second-generation cephalosporins. According to our old results, susceptibility to cefuroxime at the start of 2000 was noted in about 97% strain, and around 65% before 2018 [44]. The predominant mechanism of resistance found in 98.6% of the strains involved BRO-1 β-lactamases; this mechanism was mostly associated with high resistance to penicillins without inhibitors, first-generation cephalosporins, and, to a lesser extent, second-generation cephalosporins. Remarkably high susceptibility to other tested antimicrobials, except a few strains demonstrating resistance to trimethoprim-sulfamethoxazole, was determined. Both BRO-1 and BRO-2 enzymes are readily inactivated by β-lactamase inhibitors, and the isolates are still susceptible to amoxicillin in combination with clavulanic acid. Many studies noted that routinely administered therapy consisting of a combination of aminopenicillins and β-lactamase inhibitors is usually sufficient [13,16,45,46]. *M. catarrhalis* is generally considered to have high sensitivity to many antibiotics. Authors from different regions reported that *M. catarrhalis* was full susceptibility to amoxicillin/clavulanic acid, cefotaxime, and moxifloxacin [39,40,47]. According to other reports, *M. catarrhalis* appeared extremely susceptible to macrolide antibiotics, erythromycin, and rokitamycin [39,40,48]. Susceptibility of all tested strains to azithromycin, doxycycline, co-trimoxazole was also reported [39,40,44,49].

In a study from Hungary, non-susceptibility to macrolides and trimethoprim-sulfamethoxazole was reported at less than 5%, with low MIC values, and single resistant strains to levofloxacin were reported [44]. Complicated therapy and antimicrobial resistance have been described in Asian countries, with strains resistant to trimethoprim/sulfamethoxazole, levofloxacin, ciprofloxacin, erythromycin, gentamicin, clarithromycin, telithromycin, cefotaxime, or chloramphenicol reported [40,50,51,52]. There are increasing reports of strains with low non-susceptibility or resistance to tetracycline, and tetracycline-resistant strains were isolated more frequently than strains resistant to other antibiotics [46,51].

The serotype distribution of *M. catarrhalis* confirmed the prevalence of serotype A over the years. In an earlier study of Bulgarian isolates of *M. catarrhalis* from patients with various respiratory infections and different ages, the Bulgarian strains mainly belonged to serotype A [21]. The results of this study confirmed that serotype A is still the leading pathogen in our country. We did not observe an association among the serotypes, diagnosis, and specific clonal complexes because most strains belong to the same serotype.

The examined *M. catarrhalis* population revealed various genetic lineages. Despite this diversity, one main clonal complex denoted CC224 and other small clusters, including CC141, CC184, and CC449, were predominant. Two likely primary founders were identified: ST224 and ST141. The major CC224 was also identified in studies from China and was reported to be susceptible to macrolides [53]. Our CC224 strains showed the same susceptibility, with even more susceptibility to all tested antibiotics. We did not observe a correlation for CC224 with the age of the patients or the diagnosis. Both strains recovered from children and adult specimens with upper and lower respiratory tract infections showed genetic relatedness with CC224. 

CC141 comprised four different STs from susceptible strains. ST64 was a triple locus variant of ST141 reported in other studies as an ST resistant to azithromycin, erythromycin, and cefuroxime [50,54]. Apart from CC141, ST215 is found in our population and is also frequently identified in Asia with the same multidrug resistance reported in Chinese studies [50].

CC449 is successfully circulated in our country and has been described in reports from Asia as a purely macrolide-resistant clonal complex. In our country, all ST449 strains showed high susceptibility, but it is alarming that the same well-distributed cluster has been represented predominantly by resistance strains in other countries. We may expect strains from this cluster or CC141 to evolve in macrolide-resistant isolates and spread these genetic lineages to our geographic area. *M. catarrhalis* is a genetically heterogeneous species from which successful clones occasionally proliferate. The expansion of such competitive types has also been documented during distinct periods and in particular geographic regions [55,56,57]. Frequent horizontal gene transfer seems possible [58,59], and new phenotypic markers could be acquired through cross-species gene acquisition.

Other investigations cited the distribution of CC363, which comprised resistant *M. catarrhalis* strains to cefaclor and azithromycin; however, it is still not present in our area [54,55].

Both ST184 and ST435 from CC184 in our population were registered in the global database (https://enterobase.warwick.ac.uk/ (accessed on 31 August 2024)) in different regions in Europe, Australia, and the USA. ST67 was also a successful spreading type found in France, Australia, and the USA. 

During our investigations, we discovered sequence types, such as ST 216 and ST 54, and other STs, each present in only a small number of two or three strains. Based on this, we anticipate the spread of these clonal complexes in the coming years. These clonal complexes consist exclusively of a single sequence type, which can happen when a specific strain undergoes clonal expansion within a population, leading to the dominance of that particular sequence type in the population’s structure.

The epidemiological studies of *M. catarrhalis* are still rare and insufficient in understanding how these lineages split and subsequently expanded in the human population. 

## 4. Materials and Methods

### 4.1. Specimen Collection

Specimens were collected from patients of all age groups who had upper and lower respiratory tract infections between October and May of 2023–2024. Clinical strains of *M. catarrhalis* were isolated from nasal and nasopharyngeal swabs, punctures from ears and sinuses that were taken from children based on their clinical symptoms after a physical examination, and sputum samples collected from the adult patients only. The isolates were identified using conventional microbiological tests, including typical Gram morphology; positive tests for catalase, oxidase, and indoxyl acetate esterase reactions; the hockey puck sign; and assessments using the semiautomatic system Crystal NH BBL (Beckton Dickinson, Kelberg, Germany).

### 4.2. Antimicrobial Susceptibility Testing

Antimicrobial susceptibility testing was performed phenotypically with amoxicillin/clavulanic acid, 2/1 μg; cefotaxime 5 μg; cefuroxime 30 μg, erythromycin 15 μg; tetracycline 30 μg; chloramphenicol 30 μg; levofloxacin, 5 μg; and trimethoprim-sulfamethoxazole 30 μg (all from Mast Group Ltd., Reinfeld, Germany). European Committee on Antimicrobial Susceptibility Testing (EUCAST 2024) breakpoints were used to assess susceptibility and resistance. Production of β-lactamases was estimated using the Cefinase disc method (Beckton Dickinson, Kelberg, Germany). The primers and conditions for PCR detection of BRO-1 and BRO-2 genes were previously described [21].

### 4.3. Serotyping

Monoclonal antibodies (MAbs) against the three LOS chemotypes A, B, and C used for serotyping with an immunofluorescence method in this work were obtained in our laboratory by hybridoma technology as previously described [28]. The serotypes share a common core terminal unit, causing cross-reactivity among the three serotypes. More frequent cross-reactivity between A and C serotypes is possible potentially due to the presence of larger similar regions and common epitopes, which could provide a protective effect of antibodies against both serotypes A and C [28].

### 4.4. DNA Extraction and PCR Amplifications

Total DNA was extracted from pure cultures of *M. catarrhalis* strains using the extraction kit (DNA-Sorb-A DNA extraction kit, Sacace Biotechnologies, Como, Italy) in accordance with the manufacturer’s instructions. The extracted DNA was stored at −70 °C.

The PCR amplifications was done with 5 µL of DNA template in a 25 µL reaction mixture containing 1 µL of each primer and 10 µL 2 × Prime Taq Premix (GenetBio, Daejeon, Republic of Korea). Thermocycling was performed on the GTQ-cycler 96 (Hain lifesciences, Nehren, Germany) with PCR cycling conditions consisting of an initial denaturation at 94 °C for 4 min, followed by 35 cycles at 94 °C for 30 s, 52 °C for 30 s, and 72 °C for 30 s for all genes, except *glyRS* (58 °C) and *adk* (54 °C). The final extension was at 72 °C for 10 min. The primer sequences of the housekeeping genes are listed in Table 3.

### 4.5. Multilocus Sequence Typing (MLST) of M. catarrhalis

The PCR products were purified using the Exo-CIP™ Rapid PCR-Cleanup kit (New England Biolabs, Ipswich, MA, USA) before sequencing. Nucleotide sequencing of both strands of the PCR amplicon was performed using an ABI 3500 xl Genetic Analyzer (Applied Biosystems, Waltham, MA, USA). MLST was used for population analysis and monitoring the distribution of *M. catarrhalis* genetic lineages in our geographic area. We performed sequencing of eight “housekeeping genes”: *ppa* (pyrophosphate phospho-hydrolase), *efp* (elongation factor P), *fumC* (fumarate hydratase), *trpE* (anthranilate synthase component I), *mutY* (adenine glycosylase), *adk* (adenylate kinase), *abcZ* (ATP-binding protein), and *glyRS* (glycyl-tRNA synthetase beta subunit). We used the protocol for PCR amplification of the internal fragments of the housekeeping genes of *M. catarrhalis* described at https://enterobase.readthedocs.io/en/latest/mlst/mlst-legacy-info-mcatarrhalis.html (accessed on 31 August 2024).

After sequencing, we determined the allelic profile of each studied isolate and the sequence type (ST) on the MLST website using the Achtman scheme.

The obtained STs enabled us to analyze the genetic relationships between the isolates. A high number of identical locus variants indicates a higher degree of genetic relatedness. Within one clonal complex (CC), STs with single locus variants (SLVs), double locus variants (DLVs), or triple locus variants (TLVs) were identified. By identifying the clonal structure of the studied population, we uncovered the primary ancestral type and the organization of allelic variants in CCs based on their genetic similarity.

All clonal complexes were compared and analyzed for genetic relatedness to reference circulating clones in the international database (https://enterobase.warwick.ac.uk/species/index/mcatarrhalis (accessed on 31 August 2024)). The diagram of clonality was constructed using Phyloviz 2.0.

## 5. Conclusions

The studied population revealed that susceptible *M. catarrhalis* strains, predominantly serotype A, were isolated from children and adults with upper and lower respiratory tract infections. The analyzed genetic lineages were diverse but showed the predominance of two main clusters, CC224 and CC141, which encompassed sequence types distributed in other regions and showed multidrug resistance. These data give us reason to assume that resistant strains from the same genetic lines could also spread to our country. Therefore, ongoing epidemiological monitoring of successfully circulating clones and the implementation of adequate antibiotic policies should continue to be the subject of future studies and analyses.

## Figures and Tables

**Table 1:** Distribution of patient age, diagnosis, and serotypes of *M. catarrhalis* strains isolated during the winter–spring season of 2024 in Bulgaria.

	Age ^1^	Diagnosis ^2^			Serotype		
Children	0–11 y n = 51 (69.9%)	**URTI**	**AOM**	**LRTI**	**A**	**B**	**C**
		43 (58.9%)	8 (11.0%)	-	46 (63.0%)	4 (5.5%)	1 (1.4%)
Adults	50–74 y n = 22 (30.1%)	3 (4.1%)	-	19 (26.0%)	20(27.4%)	1 (1.4%)	1 (1.4%)
Total n (100%)	73 (100%)	46 (63.0%)	8 (11.0%)	19 (26.0%)	66 (90.4%)	5 (6.8%)	2 (2.8%)

^1^ Age—All patients were categorized into two age groups. There were no collected samples from patients between 11 and 50 years old. ^2^ Diagnosis: URTI—upper respiratory tract infection, including rhinopharyngitis, rhinosinusitis, and adenoiditis; AOM—acute otitis media; LRTI— lower respiratory tract infection, including COPD (chronic obstructive pulmonary disease) and bronchitis.

**Table 2:** Phenotypic and genotypic characteristics of 73 *M. catarrhalis* isolates recovered from children and adults in Bulgaria during the winter–spring season of 2023–2024.

CC ^1^	Strain	Age	Gender ^2^	Diagnosis ^3^	Sample ^4^	Serotype	*abcZ*	*adk*	*efp*	*fumC*	*glyRS*	*mutY*	*ppa*	*trpE*	ST ^5^
CC141	57/1379	50	f	Bronchitis chr	Sputum	A	2	17	12	2	3	3	3	2	64
	557	2	f	Rhinophar	Nph	A	2	6	2	2	20	3	3	2	141
	8080	2	f	Rhinophar	Nph	A	2	6	2	2	20	3	3	2	141
	1353	4	f	Adenoiditis	Nph	A	2	6	2	2	20	3	3	2	141
	20/1321	3	m	Adenoiditis	Nph	A	2	9	2	2	20	3	3	2	84
	58/1558	1	m	Rhinophar	Nph	A	8	6	3	2	20	26	3	2	388
CC224	77	1	f	Rhinophar	Nph	A	53	3	2	3	57	3	3	2	224
	17/179	5	m	Adenoiditis	Nph	A	53	3	2	3	57	3	3	2	224
	61	3	m	Adenoiditis	Nph	A	53	3	2	3	57	3	3	2	224
	54/847	5	m	Rhinophar	Nph	A	53	3	2	3	57	3	3	2	224
	18	6	f	Rhinophar	Nph	A	53	3	2	3	57	3	3	2	224
	711	6	f	Rhinophar	Nph	A	53	3	2	3	57	3	3	2	224
	555	71	m	COPD	Sputum	A	53	3	2	3	57	3	3	2	224
	608	61	m	Bronchitis chr	Sputum	A	53	3	2	3	57	3	3	2	224
	26/53K	71	m	COPD	Sputum	A	53	3	2	3	57	3	3	2	224
	1843	63	m	COPD	Sputum	A	53	3	59	3	57	3	3	2	589
	109,316	68	m	COPD	Sputum	A	3	3	2	3	57	67	3	2	408
	K978	5	m	Rhinophar	Nph	A	3	3	2	9	57	3	3	2	227
	166	4	m	Adenoiditis	Nph	A	3	3	2	9	57	3	3	2	227
	3270	2	m	Rhinophar	Nph	A	28	3	2	3	31	31	3	2	195
	60	67	m	COPD	Sputum	A	28	3	2	3	31	31	3	2	195
	3270	2	m	Rhinophar	Nph	A	28	3	2	3	31	31	3	2	195
CC184	5102	10	m	Rhinosinuitis	Nph	A	8	25	6	4	57	22	2	2	184
	55/980	2	f	Rhinophar	Nph	A	8	25	6	4	57	22	2	2	184
	22/6215	46	f	Rhinosinuitis	Nph	A	8	25	6	4	57	22	2	2	184
	426	5	f	AOM	MEF	A	8	25	6	4	57	22	23	2	435
	184/241	5	m	Rhinophar	Nph	A	8	25	6	4	57	22	23	2	435
	24/814	4	m	AOM	MEF	B	8	25	6	4	57	22	23	2	435
CC449	36/3468	6	m	Rhinophar	Nph	A	3	3	2	2	17	15	8	2	449
	37/8657	2	f	Rhinophar	Nph	A	3	3	2	2	17	15	8	2	449
	31/161	1	m	Rhinophar	Nph	B	3	3	2	2	17	15	8	2	449
	2826	1	m	Rhinophar	Nph	A	3	18	55	2	15	15	8	2	498
	11	2	m	Rhinophar	Nph	A	3	18	55	2	15	15	8	2	498
CC390	42/785	6	f	AOM	MEF	A	8	25	6	2	37	9	3	2	390
	785/42	6	f	AOM	MEF	B	8	25	6	2	37	9	3	2	390
	42/785	4	f	Rhinophar	Nph	A	8	25	6	2	37	9	3	2	390
	3522	3	m	Adenoiditis	Nph	A	8	3	6	2	17	9	17	2	197
CC67	184	11	m	Rhinosinuitis	Nph	A	2	17	2	3	20	2	2	2	67
	571	60	m	COPD	Sputum	A	2	2	111	3	2	2	2	2	928
ST3	75	3	f	Rhinophar	Nph	A	2	2	2	3	2	2	2	2	3
	1205	3	m	Rhinophar	Nph	A	2	2	2	3	2	2	2	2	3
ST54	592	2	f	Rhinophar	Nph	A	8	24	3	7	30	3	23	2	54
	353	59	m	Rhinosinuitis	Nph	A	8	24	3	7	30	3	23	2	54
	343	1	f	Rhinosinuitis	Nph	A	8	24	3	7	30	3	23	2	54
ST62	K83	3	m	AOM	MEF	C	8	26	2	3	2	22	25	2	62
	1096	4	m	AOM	Nph	A	8	26	2	3	2	22	25	2	62
ST183	43/1680	2	m	AOM	Nph	A	9	3	2	3	37	19	9	2	183
	141	10	m	Rhinosinuitis	Nph	A	9	3	2	3	37	19	9	2	183
ST215	K1092	1	f	Rhinophar	Nph	A	2	2	2	9	2	18	17	2	215
	K1097	3	m	AOM	Nph	A	2	2	2	9	2	18	17	2	215
ST216	10,838	74	m	COPD	Sputum	A	25	18	3	4	6	9	3	2	216
	56/1497	2	f	Rhinophar	Nph	A	25	18	3	4	6	9	3	2	216
	39/4638	51	f	Bronchitis chr	Sputum	A	25	18	3	4	6	9	3	2	216
ST436	8083	3	m	AOM	Nph	B	27	18	2	2	10	9	9	2	436
	32/160	65	m	COPD	Sputum	A	27	18	2	2	10	9	9	2	436
ST540	22/471	53	m	COPD	Sputum	B	8	17	20	3	128	3	17	2	540
	28/3220	64	m	Bronchitis chr	Sputum	A	8	17	20	3	128	3	17	2	540
ST952	40/348	4	f	Rhinopharyn	Nph	A	157	2	2	9	21	9	3	2	952
	22/5205M	4	f	Rhinophar	Nph	A	157	2	2	9	21	9	3	2	952
ST1022	81	6	f	AOM	Nph	A	3	3	3	4	3	28	3	2	1022
	14	2	m	Rhinophar	Nph	A	3	3	3	4	3	28	3	2	1022
STN	K511	4	f	Adenoiditis	Nph	A	3	9	2	9	129	8	9	5	STN
	512	3	m	Otitis media	MEF	A	3	9	2	9	129	8	9	2	STN
Singletons	69,215	2	m	Rhinophar	Nph	A	3	3	3	4	3	3	3	2	4
	3034	4	m	Adenoiditis	Nph	A	3	18	6	3	31	15	17	2	56
	64	57	m	COPD	Sputum	A	8	26	2	3	2	22	25	2	62
	30/867	4	f	Otitis media	MEF	A	2	17	12	2	3	3	3	2	64
	34/3907	54	f	Bronchitis chr	Sputum	A	29	2	2	3	34	29	21	2	74
	72AI	65	m	Bronchitis chr	Sputum	C	36	6	12	2	50	41	3	2	126
	17	5	f	Adenoiditis	Nph	A	8	17	6	3	27	3	3	2	164
	3168	4	m	Rhinophar	Nph	A	2	17	12	3	3	3	3	2	338
	80	55	m	COPD	Sputum	A	2	17	2	4	107	89	3	2	437
	25/91K	74	m	Bronchitis chr	Sputum	A	108	3	2	3	31	31	21	2	618

^1^ CC—clonal complex. ^2^ Gender: f—female, m—male. ^3^ Diagnosis: Bronchitis chr—chronical bronchitis, Rhinophar—rhinopharyngitis, AOM—acute otitis media, COPD—chronic obstructive pulmonary disease. ^4^ Sample: Nph—nasopharynx, MEF—middle ear fluid. ^5^ ST—sequence type.

**Table 3:** Primer sets for PCR amplification of eight *M. catarrhalis* housekeeping genes.

Gene	Primer Set	Base Pairs
*glyRS*	F 5′-GCACCGAAGAGTTGCCACCA-3′ R 5′-ACGCAACGGGCAAATCCACC-3′	762 bp
*ppa*	F 5′-AATAAAATTCTAGATGCTGGC-3′ R 5′-ACTTATTGCTCTGTCCAGCG-3′	523 bp
*efp*	F 5′-CTCTGATTGACAACTGGCAGG-3′ R 5′-GATATTCGCCAGTACGCG-3′	582 bp
*fumC*	F 5′-GGGCGGTACAGCAGTCGGCAC-3′ R 5′-CTCATCAAATTCAGCTTCAG-3′	675 bp
*trpE*	F 5′-TTATCCCGCATCGAAAATGG-3′ R 5′-GGTTTCATCCCATTCAGCC-3′	545 bp
*mutY*	F 5′-GGCAATACCATCATCAGCCG-3′ R 5′-GGTAACTGACTTTGAACGCC-3′	609 bp
*adk*	F 5′-GGCATTCCTCAAATCTCAAC-3′ R 5′-GATGGGCTTTATTGTCAAATG-3′	631 bp
*abcZ*	F 5′-ACATGCTGATGATGGTGAG-3′ R 5′-CACTGGCAAGTTCAAGCGC-3′	610 bp

The same primers were used for the following MLST procedure, except the primer sets for the following three genes: *glyRS*: F5′-GCACCGAAGAGTTGCCACCA-3′ and R5′-ATATCGGCTTGACGCTGATC-3′; *fumC*: F5′-GCTGTCAAAGTCGCTAAAG-3′ and R5′-CTCATCAAATTCAGCTTCAG-3′, and *mutY*: F5′-TATGCTGTGTGGGTATCTG-3′ and R5′-GGTAACTGACTTTGAACGCC-3′.

## Data Availability

Data is contained within the article.

## References

[1] Karalus R., Campagnari A. (2000). *Moraxella catarrhalis*: A review of an important human mucosal pathogen. Microbes Infect..

[2] Deng W.J., Zhang J.F., Li P.Y., Zhou J.L., Yao Z.J., Ye X.H. (2022). Co-carriage of *Streptococcus pneumoniae* and *Moraxella catarrhalis* among preschool children and its influencing factors. Zhongguo Dang Dai Er Ke Za Zhi.

[3] Verduin C.M., Hol C., Fleer A., van Dijk H., van Belkum A. (2002). *Moraxella catarrhalis*: From emerging to established pathogen. Clin. Microbiol. Rev..

[4] Morris D.E., Osman K.L., Cleary D.W., Clarke S.C. (2022). The characterization of *Moraxella catarrhalis* carried in the general population. Microb. Genom..

[5] Aebi C. (2011). *Moraxella catarrhalis*—Pathogen or commensal?. Hot Topics in Infection and Immunity in Children VII.

[6] Goldstein E.J.C., Murphy T.F., Parameswaran G.I. (2009). *Moraxella catarrhalis*, a human respiratory tract pathogen. Clin. Infect. Dis..

[7] Gupta N., Arora S., Kundra S. (2011). *Moraxella catarrhalis* as a respiratory pathogen. Indian J. Pathol. Microbiol..

[8] Van Hare G.F., Shurin P.A. (1987). The increasing importance of *Branhamella catarrhalis* in respiratory infections. Pediatr. Infect. Dis. J..

[9] Mbaki N., Rikitomi N., Nagatake T., Matsumoto K., Tohoku J. (1987). Correlation between *Branhamella catarrhalis* adherence to oropharyngeal cells and seasonal incidence of lower respiratory tract infections. Tohoku J. Exp. Med..

[10] Wilkinson T.M.A., Aris E., Bourne S., Clarke S.C., Peeters M., Pascal T.G., Schoonbroodt S., Tuck A.C., Kim V., Ostridge K. (2017). A prospective, observational cohort study of the seasonal dynamics of airway pathogens in the aetiology of exacerbations in COPD. Thorax.

[11] Marchisio P., Gironi S., Esposito S., Schito G.C., Mannelli S., Principi N. (2001). Seasonal variations in nasopharyngeal carriage of respiratory pathogens in healthy Italian children attending day-care centres or schools. J. Med. Microbiol..

[12] Venekamp R.P., Damoiseaux R.A., Schilder A.G. (2017). Acute Otitis Media in Children. Am. Fam. Physician.

[13] El Feghaly R.E., Nedved A., Katz S.E., Frost H.M. (2023). New insights into the treatment of acute otitis media. Expert Rev. Anti-Infect. Ther..

[14] Zhang X.B., Wu X., Nong G.M. (2020). Update on protracted bacterial bronchitis in children. Ital. J. Pediatr..

[15] Shaikh N., Hoberman A., Shope T.R., Jeong J.H., Kurs-Lasky M., Martin J.M., Bhatnagar S., Muniz G.B., Block S.L., Andrasko M. (2023). Identifying Children Likely to Benefit from Antibiotics for Acute Sinusitis: A Randomized Clinical Trial. JAMA.

[16] Nawa M., Mwansa J., Mwaba J., Kaonga P., Mukubesa A.N., Simuyandi M., Chisenga C.C., Alabi P., Mwananyanda L., Thea D.M. (2022). Microbiologic and virulence characteristics of *Moraxella catarrhalis* isolates from Zambian children presenting with acute pneumonia. Pediatr. Pulmonol..

[17] Siegel H., Lang S., Maier P., Reinhard T. (2024). Bacterial Conjunctivitis: Current Aspects of Diagnosis and Therapy. Klin. Monbl. Augenheilkd..

[18] Perez A.C., Murphy T.F. (2019). Potential impact of a *Moraxella catarrhalis* vaccine in COPD. Vaccine.

[19] Wiegers H.M.G., van Nijen L., van Woensel J.B.M., Bem R.A., de Jong M.D., Calis J.C.J. (2019). Bacterial co-infection of the respiratory tract in ventilated children with bronchiolitis; a retrospective cohort study. BMC Infect. Dis..

[20] Hirai J., Kinjo T., Koga T., Haranaga S., Motonaga E., Fujita J. (2020). Clinical characteristics of community-acquired pneumonia due to *Moraxella catarrhalis* in adults: A retrospective single-centre study. BMC Infect. Dis..

[21] Mitov I.G., Gergova R.T., Ouzounova-Raykova V.V. (2010). Distribution of genes encoding virulence factors *ompB2*, *ompCD*, *ompE*, β-lactamase and serotype in pathogenic and colonizing strains of *Moraxella catarrhalis*. Arch. Med. Res..

[22] Harb H., Al-Obaidi H., Irannejad K., Bagheri F.A. (2024). Unique Case of *Moraxella catarrhalis* Meningitis Following Neurosurgical Intervention. Cureus.

[23] Ioannou P., Alexakis K., Baliou S., Kofteridis D.P. (2022). Infective Endocarditis by Moraxella Species: A Systematic Review. J. Clin. Med..

[24] Siwakoti S., Bajracharya S., Adhikaree N., Sah R., Rajbhandari R.S., Khanal B. (2019). Early-Onset Neonatal Meningitis Caused by an Unusual Pathogen-Moraxella catarrhalis. Case Rep. Pediatr..

[25] Daoud A., Abuekteish F., Masaadeh H. (1996). Neonatal meningitis due to *Moraxella catarrhalis* and review of the literature. Ann. Trop. Paediatr..

[26] Kobayashi Y. (2000). Bacteremic *Moraxella catarrhalis* pneumonia. J. Infect. Chemother..

[27] Apisarnthanarak A., Mundy L.M. (2005). Etiology of community-acquired pneumonia. Clin. Chest Med..

[28] Gergova R.T., Iankov I.D., Haralambieva I.H., Mitov I.G. (2007). Bactericidal monoclonal antibody against *Moraxella catarrhalis* lipooligosaccharide cross-reacts with *Haemophilus* spp.. Curr. Microbiol..

[29] de Vries S.P., Bootsma H.J., Hays J.P., Hermans P.W. (2009). Molecular aspects of *Moraxella catarrhalis* pathogenesis. Microbiol. Mol. Biol. Rev..

[30] Rikitomi N., Ahmed K., Nagatake T. (1997). *Moraxella (Branhamella) catarrhalis* adherence to human bronchial and oropharyngeal cells: The role of adherence in lower respiratory tract infections. Microbiol. Immunol..

[31] Reddy M.S., Murphy T.F., Faden H.S., Bernstein J.M. (1997). Middle ear mucin glycoprotein: Purification and interaction with nontypable Haemophilus influenzae and *Moraxella catarrhalis*. Otolaryngol.—Head Neck Surg..

[32] Luke N.R., Howlett A.J., Shao J., Campagnari A.A. (2004). Expression of type IV pili by *Moraxella catarrhalis* is essential for natural competence and is affected by iron limitation. Infect. Immun..

[33] Steimle A., Autenrieth I.B., Frick J.S. (2016). Structure and function: Lipid A modifications in commensals and pathogens. Int. J. Med. Microbiol..

[34] Xiao X., Sankaranarayanan K., Khosla C. (2017). Biosynthesis and structure-activity relationships of the lipid a family of glycolipids. Curr. Opin. Chem. Biol..

[35] Gao Y., Lee J., Widmalm G., Im W. (2020). Preferred conformations of lipooligosaccharides and oligosaccharides of *Moraxella catarrhalis*. Glycobiology.

[36] McGregor K., Chang B.J., Mee B.J., Riley T.V. (1998). *Moraxella catarrhalis*: Clinical significance, antimicrobial susceptibility and BRO beta-lactamases. Eur. J. Clin. Microbiol. Infect. Dis..

[37] Khan M.A., Northwood J.B., Levy F., Verhaegh S.J., Farrell D.J., Van Belkum A., Hays J.P. (2010). *bro* β-lactamase and antibiotic resistances in a global cross-sectional study of *Moraxella catarrhalis* from children and adults. J. Antimicrob. Chemother..

[38] Bootsma H.J., van Dijk H., Vauterin P., Verhoef J., Mooi F.R. (2000). Genesis of BRO β-lactamase-producing *Moraxella catarrhalis*: Evidence for transformation-mediated horizontal transfer. Mol. Microbiol..

[39] Bristy S.A., Hossain M.A., Hasan M.I., Mahmud S.M.H., Moni M.A., Rahman M.H. (2023). An integrated complete-genome sequencing and systems biology approach to predict antimicrobial resistance genes in the virulent bacterial strains of *Moraxella catarrhalis*. Brief. Funct. Genom..

[40] Zhang Z., Yang Z., Xiang X., Liao P., Niu C. (2022). Mutation of TonB-Dependent Receptor Encoding Gene MCR_0492 Potentially Associates with Macrolides Resistance in *Moraxella catarrhalis* Isolates. Infect. Drug Resist..

[41] Sánchez Arlegui A., Del Arco Rodríguez J., De Velasco Vázquez X., Gallego Rodrigo M., Gangoiti I., Mintegi S. (2024). Bacterial pathogens and antimicrobial resistance in acute otitis media. An. Pediatr. Engl. Ed..

[42] Ngo C.C., Massa H.M., Thornton R.B., Cripps A.W. (2016). Predominant Bacteria Detected from the Middle Ear Fluid of Children Experiencing Otitis Media: A Systematic Review. PLoS ONE.

[43] Wood G.M., Johnson B.C., McCormack J.G. (1996). *Moraxella catarrhalis*: Pathogenic significance in respiratory tract infections treated by community practitioners. Clin. Infect. Dis..

[44] Gergova R., Markovska R. (2020). Antimicrobial resistance of Bulgarian isolates *Moraxella catarrhalis* during the period 1999–2018. J. IMAB.

[45] Hare K.M., Seib K.L., Chang A.B., Harris T.M., Spargo J.C., Smith-Vaughan H.C. (2019). Antimicrobial susceptibility and impact of macrolide antibiotics on *Moraxella catarrhalis* in the upper and lower airways of children with chronic endobronchial suppuration. J. Med. Microbiol..

[46] Król-Turmińska K., Olender A., Bogut A. (2020). Tetracycline resistance in *Moraxella catarrhalis* clinical strains isolated in Poland. New Microbiol..

[47] Kovács E., Sahin-Tóth J., Tóthpál A., van der Linden M., Tirczka T., Dobay O. (2020). Co-carriage of *Staphylococcus aureus*, *Streptococcus pneumoniae*, *Haemophilus influenzae* and *Moraxella catarrhalis* among three different age categories of children in Hungary. PLoS ONE.

[48] Chen L., Zhang Y.H., Wang S., Zhang Y., Huang T., Cai Y. (2017). Prediction and analysis of essential genes using the enrichments of gene ontology and KEGG pathways. PLoS ONE.

[49] Bandet T., Whitehead S., Blondel-Hill E., Wagner K., Cheeptham N. (2014). Susceptibility of clinical *Moraxella catarrhalis* isolates in British Columbia to six empirically prescribed antibiotic agents. Can. J. Infect. Dis. Med. Microbiol..

[50] Zhao N., Ren H., Deng J., Du Y., Li Q., Zhou P., Zhou H., Jiang X., Qin T. (2022). Genotypic and Phenotypic Characteristics of *Moraxella catarrhalis* from Patients and Healthy Asymptomatic Participants among Preschool Children. Pathogens.

[51] Flamm R.K., Sader H.S., Farrell D.J., Jones R.N. (2012). Macrolide and tetracycline resistance among *Moraxella catarrhalis* isolates from 2009 to 2011. Diagn. Microbiol. Infect. Dis..

[52] Raveendran S., Kumar G., Sivanandan R.N., Dias M. (2020). *Moraxella catarrhalis*: A Cause of Concern with Emerging Resistance and Presence of BRO Beta-Lactamase Gene-Report from a Tertiary Care Hospital in South India. Int. J. Microbiol..

[53] Liu Y.L., Ding R., Jia X.M., Huang J.J., Yu S., Chan H.T., Li W., Mao L.L., Zhang L., Zhang X.Y. (2022). Correlation of *Moraxella catarrhalis* macrolide susceptibility with the ability to adhere and invade human respiratory epithelial cells. Emerg. Microbes Infect..

[54] Liu Y.L., Xiao M., Cheng J.W., Xu H.P., Xu Z.P., Ye S., Zhang W.J., Kudinha T., Kong F., Xu Y.C. (2017). *Moraxella catarrhalis* Macrolide-Resistant isolates Are highly concentrated in Two MLST clonal complexes -CCN10 and CC363. Front. Microbiol..

[55] Du Y., Zhou H., Wang F., Liang S., Cheng L., Du X., Pang F., Tian J., Kan B., Xu J. (2017). Multilocus sequence typing-based analysis of *Moraxella catarrhalis* population structure reveals clonal spreading of drug-resistant strains isolated from childhood pneumonia. Infect. Genet. Evol..

[56] Qin L., Masaki H., Gotoh K., Furumoto A., Terada M., Watanabe K., Watanabe H. (2009). Molecular epidemiological study of *Moraxella catarrhalis* isolated from nosocomial respiratory infection patients in a community hospital in Japan. Intern. Med..

[57] Hansen K., YambaYamba L., Wasserstrom L., Rünow E., Göransson T., Nilsson A., Ahl J., Riesbeck K. (2023). Exploring the microbial landscape: Uncovering the pathogens associated with community-acquired pneumonia in hospitalized patients. Front. Public Health.

[58] Blakeway L.V., Tan A., Lappan R., Ariff A., Pickering J.L., Peacock C.S., Blyth C.C., Kahler C.M., Chang B.J., Lehmann D. (2018). *Moraxella catarrhalis* Restriction-Modification Systems Are Associated with Phylogenetic Lineage and Disease. Genome Biol. Evol..

[59] Earl J.P., de Vries S.P., Ahmed A., Powell E., Schultz M.P., Hermans P.W., Hill D.J., Zhou Z., Constantinidou C.I., Hu F.Z. (2016). Comparative Genomic Analyses of the *Moraxella catarrhalis* Serosensitive and Seroresistant Lineages Demonstrate Their Independent Evolution. Genome Biol. Evol..

